# Neuronal glycolysis: focus on developmental morphogenesis and localized subcellular functions

**DOI:** 10.1080/19420889.2024.2343532

**Published:** 2024-04-21

**Authors:** Gianluca Gallo

**Affiliations:** Department of Neural Sciences, Shriners Pediatric Research Center, Lewis Katz School of Medicine at Temple University, Philadelphia, PA, USA

**Keywords:** Bioenergetics, axon, actin, glycolysis, mitochondria

## Abstract

Glycolysis is a metabolic pathway that directly generates adenosine triphosphate (ATP), provides metabolic intermediates for anabolism, and supports mitochondrial oxidative phosphorylation. This review addresses recent advances in our understanding of the functions of neuronal glycolysis during the development of neuronal morphogenesis, focusing on the emergent concept that neuronal glycolysis serves local subcellular bioenergetic roles in maintaining neuronal function. The current evidence indicates that glycolysis is subcellularly targeted to specific organelles and molecular machinery to locally supply bioenergetic support for defined subcellular mechanisms underlying neuronal morphogenesis (i.e. axon extension, axon retraction and axonal transport). Thus, the concept of glycolysis as a “housekeeping” mechanism in neurons would benefit revision and future work aim to further define its subcellular functions at varied developmental stages.

Glycolysis is an ancient intracellular bioenergetic process that utilizes glucose to generate adenosine triphosphate (ATP) and provides intermediate metabolites for the anabolism of amino acids, nucleotides and triglycerides (Figure 1) [1]. Pyruvate derived from the last step of glycolysis is then fed into the tricarboxylic acid cycle to fuel mitochondrial oxidative phosphorylation. Glycolysis generates two ATPs per glucose molecule in contrast to oxidative phosphorylation that generates 30–32 ATP. While the yield of ATP per glucose is greatest for oxidative phosphorylation, the rate (i.e., ATP/unit time) of ATP production is equivalent to or greater for glycolysis relative to oxidative phosphorylation [2]. The roles of mitochondria and oxidative phosphorylation have been thoroughly studied in neurons. In contrast, the functions of glycolysis in neuronal biology have not received as much attention. This focused review addresses the emergent understanding of subcellularly defined roles in neuronal glycolysis with emphasis on neuronal development.

**Figure 1.**
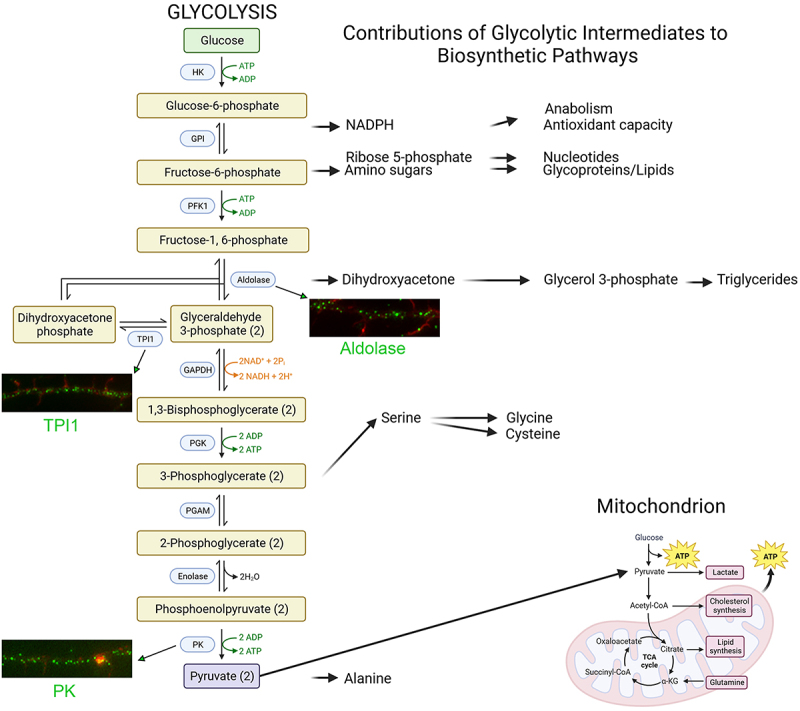
Glycolysis. On the left, glycolytic enzymes (blue boxes) and their products are shown representing the 10 steps of glycolysis. Steps using or generating ATP are denoted in green. On the right, the contributions of glycolysis to biosynthetic pathways. Pyruvate feeds into the TCA cycle and supports mitochondrial oxidative phosphorylation (bottom right). Examples of the distribution of select endogenous glycolysis enzymes detected through immunocytochemistry along the axons of embryonic chicken sensory neurons are shown. Enzymes are labeled in green and actin filaments in red. The examples shown are from our laboratory. Figure prepared using Biorender.com.

## Bioenergetics of the developing nervous system: a brief overview

Glucose in the central nervous system is derived from the vasculature. Vascularization of the mouse cerebral cortex is commenced around embryonic day (E) 10 and continues to develop in complexity and coverage of the nervous tissue until postnatal day 7 [3]. At E10 neural progenitors are giving rise to the earliest migratory neuronal populations that will then undergo full differentiation upon reaching their perspective cortical layer and start developing synaptic networks [4,5]. Up to E17 the vast majority of cells in the developing cortices are neurons, while astrocyte development occurs largely in the postnatal period [4,5]. Neuronal development is an energy-intensive process as the neurons form dendrites and axons that collectively vastly increase cellular volume. The absence of astrocytes during the major period of neuronal development and establishment of cerebral vasculature obviates the likelihood of an astrocyte-based lactate shuttle [6–9] being operative supporting the bioenergetic needs of developing neurons. Consistently, neural progenitors and early developing neurons rely heavily on neuronal glycolysis for ATP generation and likely the related anabolic functions of glycolysis [10]. Hippocampal neurons developing in vitro undergo a shift from a major reliance on glycolysis to oxidative phosphorylation as the predominant sources of ATP [11]. This switch from glycolytic prominence to an increased bioenergetic output by oxidative phosphorylation is observed in multiple neuronal types [12]. Collectively, these considerations indicate that glycolysis is a major bioenergetic source in the developing nervous system and that neuronal glycolysis is the likely predominant bioenergetic mechanism used during early neuronal development. For a more in-depth discussion of the developmental bioenergetics of neurons, readers are directed to additional reviews [12–15].

## Localization of the glycolytic apparatus in developing neurons

**Figure 2.**
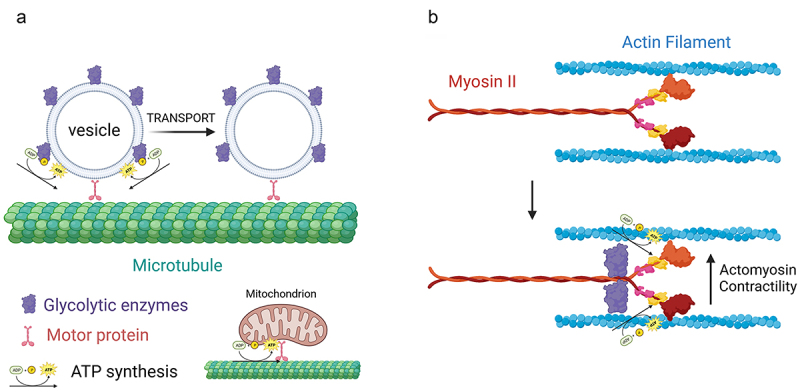
Glycolysis as a localized bioenergetic source along axons. (a) “On-board” glycolysis. Glycolytic enzymes are associated with fast transport vesicles along axons. The vesicle associated enzymes then generate ATP that is used by the vesicle associated motor proteins to move vesicles along microtubules. In contrast, mitochondria power their own transport by oxidative phosphorylation. (b) During cannabinoid induced axon retraction glycolytic enzymes are recruited to myosin II, the main ATPase mechanoenzyme that powers cellular contractility through actin filaments. The Myosin II associated glycolytic enzymes then generate ATP that is used by myosin II to power actomyosin contractility and drive axon retraction. Figure prepared using Biorender.com.

Neurons have complex morphologies characterized by an axon and multiple dendrites. During development, extending dendrites and axons are tipped by growth cones. Growth cones are dynamic structures supported by an underlying actin filament cytoskeleton. Axons and dendrites stop extending after circuit formation, although still capable of local morphologic plasticity, and synapses form at contacts between axons and dendrites. Glycolysis consists of 10 steps each mediated by a separate enzyme (Figure 1). The distribution of glycolytic enzymes in the axons developing neurons was recently assessed [16,17]. The consensus is that glycolytic enzymes are distributed as puncta along axons and in growth cones (Figure 1). The punctate distribution is considered to reflect the association of glycolytic enzymes with endomembrane compartments [18,19]. In cultured embryonic cortical neurons, glycolytic enzymes associate with signaling endosomes induced by BDNF treatment [18]. The active axonal transport of BDNF endosomes and other fast transport vesicles is in turn dependent on glycolysis and nonoxidative phosphorylation, while the latter drives mitochondrial transport (Figure 2a) [18,19]. These observations have given rise to the concept of “on-board” glycolysis serving as the bioenergetic source to power the transport of axonal vesicles, and conversely that mitochondria power their own transport. More generally, these data indicate that glycolysis, and also oxidative phosphorylation, can serve local bioenergetic functions along axons.

Glycolytic enzymes are also concentrated at synapses within established circuits [20]. The evidence to date provides strong support that local glycolysis at synapses is an important contributor to synaptic function and it is a regulated process. Inhibition of glycolysis results in failure of synaptic vesicle endocytosis, indicating a role in membrane turnover [21]. A role for glycolysis in membrane turnover is overall consistent with the association of glycolytic enzymes, with vesicles undergoing transport [18,19]. The uptake of glucose through transporter-based mechanisms is also increased at synaptic sites in response to activity [22]. Importantly, the distribution of glycolytic enzymes along axons can also be controlled. In response to energy stress, C. elegans axons locally recruit glycolytic enzymes to synaptic sites [23]. Thus, emerging evidence indicates that neuronal glycolysis is a controlled process with subcellular specificity and not a mere “housekeeping” mechanism.

The recent literature reviewed above indicates that axons have glycolytic capacity. The main themes emerging from this work are that glycolysis can serve as a localized bioenergetic source (e.g., “on-board” glycolysis for axonal vesicles) and that the distribution of glycolytic enzymes can be reconfigured on demand. Additional work is required to gain insights into the mechanism by which the distribution of glycolytic enzymes is controlled. Multiple glycolytic enzymes can bind the actin filament cytoskeleton [24]. Thus, the microtubule cytoskeleton may serve as a substratum for redistribution of glycolytic enzymes along axons through their association with axonal vesicles, and the actin cytoskeleton may serve to control the local distribution of glycolytic enzymes once targeted to a specific subcellular domain. Also, glycolytic enzymes associated with axonal vesicles may be delivered to additional membranes through vesicle fusion.

## Requirement for glycolysis in axon extension and retraction

**Figure 3.**
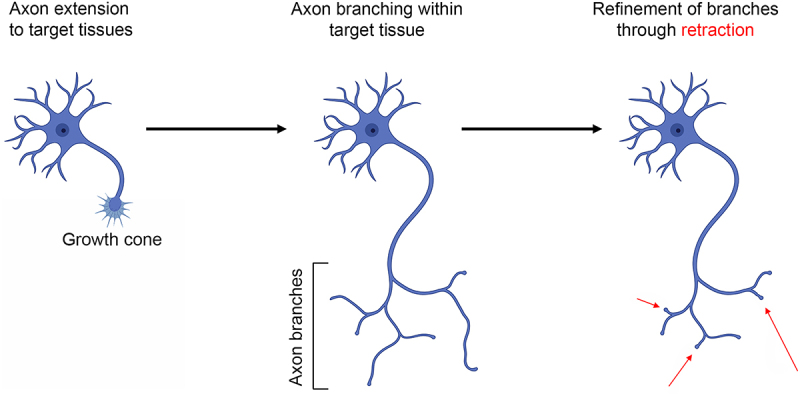
Overview of axon development. Axons initially form from the cell bodies, develop a growth cone, and extend to their target tissue. Within target tissues, or along the way, the axons generate collateral branches. Finally, during the period of refinement, supernumerary axon branches are pruned/retracted as a component of the ongoing establishment of synaptic circuitry. Figure prepared using Biorender.com.

Axons initially guide their targets but also establish branches for additional targets or within the target field (Figure 3). Axon guidance and extension is mediated by the axon’s terminus, the growth cone. During a developmental period termed refinement, supernumerary branches are subsequently retracted to give rise to the mature innervation pattern. Mitochondrial oxidative phosphorylation is necessary for embryonic and regenerative axon extension [13,15]. However, recent reports highlight the requirement for intra-axonal glycolysis in both axon extension and retraction. Glycolysis and oxidative phosphorylation are both sources of ATP in the growth cones of cultured embryonic chicken sensory neurons, and both are required to maintain axon extension [16]. Block of oxidative phosphorylation results in growth cone collapse (the loss of lamellipodia and filopodia resulting in a blunt ending). In contrast, inhibition of glycolysis causes an initial suppression of lamellipodial and filopodial protrusive activity followed by a slow decrease in surface area [16]. While inhibition of oxidative phosphorylation results in depolymerization of growth cone actin filaments, inhibition of glycolysis increases actin filament levels in conjunction with a reorganization into bundle-like structures spanning the growth cone and within the distal axon shaft [16]. Microfluidic chamber experiments, allowing specific manipulation of distal axons independent of the cell bodies, show that the effect of inhibiting glycolysis on axon extension and growth cone dynamics is local [16]. Similarly, pharmacological inhibition or disruption of the subcellular localization of the glycolytic enzyme hexokinase impairs axon elaboration in adult sensory neurons in vitro [25]. Sensory neurons of adult type-I diabetic rats exhibit impaired glycolysis and axon elaboration in vitro [26]. The effects of diabetes on glycolysis and axon extension can be reversed through treatment with the growth factor IGF-1 [27]. IGF-1 is considered to act through the positive regulation of the transcription factor CREB. Evidence for a role of glycolysis in neuronal process (neurites) extension has also been presented for embryonic cortical neurons [28]. Driving activity in cultured embryonic cortical neurons increases glucose uptake and glycolysis through upregulation of the glucose GLUT3 transporter [28]. Activity drives the expression of glycolytic genes through CREB. The upregulation of glycolysis is in turn required for the activity-driven promotion of neurite extension from cortical neurons. In vivo treatment of early postnatal rat pups with a pharmacological inhibitor of glycolysis resulted in stunted development of neurites emanating from the cell bodies of cortical neurons, likely reflective of dendrites. Collectively, these studies indicate that glycolysis is linked to axon/neurite extension through the regulation of the expression of glycolytic enzymes by CREB.

Axon retraction in response to signals during development is mediated by myosin II driven actomyosin contractility along axons [29]. Myosin II is a force generating mechanoenzyme that binds actin filaments to generate contraction. An early study on tension-driven axon retraction following detachment from culturing substrata, which is also dependent on myosin II [30], found that inhibition of glycolysis impeded the ensuing retraction [31]. A recent study significantly extended our understanding of the role of glycolysis in axon retraction [17]. Endogenous cannabinoids drive the retraction of corticofugal axons in vivo and in vitro. In vitro cannabinoids induce axon retraction through a mechanism dependent on axonal glycolysis and independent of oxidative phosphorylation. Microfluidic experiments determined that the role of glycolysis in axon retraction is mediated locally within the axon. Cannabinoid treatment did not alter axonal bioenergetics. Interestingly, through a variety of approaches, it was shown that glycolytic enzymes become recruited to myosin II following cannabinoid treatment. The redistribution of glycolytic enzymes to myosin II represents an additional venue for evidence that, as with axonal vesicles, the glycolytic apparatus is targeted to provide local bioenergetic support to specific cellular processes (Figure 2b).

## Glycolysis predominates in the cell bodies of adult neurons: implications for developing neurons

In cultured embryonic sensory neurons, the ATP/ADP ratios at the growth cone and within the cell body do not differ [32], as assessed using the live imaging ratiometric reporter PercevalHR. In contrast, ratiometric measurements of the ATP level using iATPSnFR revealed that the growth cone has nearly twice the level of ATP as the cell body [32]. This difference in ATP level between the growth cones and the cell bodies may reflect different contributions of glycolysis and oxidative phosphorylation to the local ATP level. Using a variety of approaches, glycolysis was found to contribute largely to the ATP level in the cell bodies of multiple types of nervous system neurons [33]. In the same study, glycolysis was found to be more active in cell bodies than within brain synaptosomal fractions, indicating that in adult CNS neurons there is a subcellular difference in the relative contributions of glycolysis and oxidative phosphorylation to local bioenergetics. The difference can be attributed to the predominance of the targeting of the key glycolytic enzyme pyruvate kinase M2 (PKM2) relative to PKM1 to cell bodies, which other evidence indicates preferentially drive entry of pyruvate into the tricarboxylic acid cycle and its reduction into lactate, respectively. Whether such differences in PKM isoform targeting may occur in developing neurons during morphogenesis remains to be addressed. Similarly, it remains to be determined if the subcellular prominence of glycolysis as a bioenergetic source in the cell bodies of embryonic neurons may underly the described differences in ATP level between cell bodies and growth cones. It will thus be of interest to continue the analysis of the relative contributions of glycolysis and oxidative phosphorylation to cellular physiology and the targeting of glycolytic enzymes at the subcellular level.

## Concluding statement

Investigation of the role of glycolysis in neuronal development is an emergent field. The new evidence reviewed herein indicates that our understanding of neuronal bioenergetics would be greatly expanded by continued analysis of the contributions of glycolysis to neuronal development. Importantly, recent developments indicate that bioenergetic sources (glycolysis and mitochondrial oxidative phosphorylation) likely play local roles in meeting energetic demands for specific cellular processes. An additional example of the specificity of contributions by bioenergetic sources to cellular processes is the observation that, while inhibition of oxidative phosphorylation blocks, the nerve growth factor induced phosphorylation of the kinase Akt at both major regulatory sites along axons, inhibition of glycolysis only impacts the secondary phosphorylation [34]. Principles gleaned from the study of developing neurons would likely be applicable to later stages of neuronal biology. The mature neuron differs significantly from developing neurons in terms of gene expression patterns, relative morphological stability, and pronounced synaptic activity emphasizing the role of mature neurons as components of neural circuitry. As the neuron transitions from a high glycolytic state along axons to a lower state during development, in mature neurons the contributions of glycolysis to neuronal function may exhibit highly localized functions. Consistent with this suggestion, glycolysis has been detailed to power synaptic transmission which occurs on a micron scale at synapses. It is suggested that future investigations into neuronal glycolysis would benefit from considering the hypothesis that neuronal glycolysis and likely mitochondrial oxidative phosphorylation may be contributing as a localized subcellular bioenergetic source and neither is a “powerhouse of the cell” but rather a local energy source subserving specific cellular processes.

## Data Availability

This is a review article and contains no data.
